# Vaginal microbiome and recurrent pregnancy loss

**DOI:** 10.1128/iai.00053-25

**Published:** 2025-06-30

**Authors:** Xingxing Yuan, Jiawei Gao, Ousman Bajinka, Xiaoling Feng

**Affiliations:** 1 First Clinical Medical College, Heilongjiang University of Chinese Medicine 118437, Harbin, China; 2 Department of Medicine, Heilongjiang Academy of Traditional Chinese Medicine 665996 , Harbin, China; 3 Medical Science and Technology Innovation Center, Shandong First Medical University and Shandong Academy of Medical Sciences 518873, Jinan, China; 4 Department of Gynecology, The First Affiliated Hospital of Heilongjiang University of Chinese Medicine 118437, Harbin, China; University of California Merced, Merced, California, USA

**Keywords:** recurrent pregnancy loss, vaginal microbiome, lactobacilli, metabolites, immune response

## Abstract

The joy of every mother is to survive a healthy pregnancy and give birth to a healthy baby. However, until today, many couples are finding it difficult to welcome a baby. Among the factors that cause infertility and recurrent pregnancy loss (RPL) is the microbiome composition that inhabits the vaginal space. These microbiomes occupying the vaginal space play a role in balancing acids, pH, and metabolites to ensure a healthy vaginal environment that can prevent pregnancy loss. What is even more evident is that these microbiomes, when dominated by *Lactobacillus* spp.*,* prevent the growth of vaginal pathogens and reduce the risk of developing drug resistance. Although there is compelling evidence centered on the vaginal microbiome in promoting a healthy vagina, RPL is attributed to their altered or reduced *Lactobacillus* spp. While there are discrepancies in the literature, this review aimed to summarize the recent findings on vaginal microbiome and RPL. In addition, this mini review further revealed vaginal microbiota as biomarkers that can predict a healthy vagina and the risk of vaginal microbiome causing RPL. In addition, the immune response and metabolite changes in vaginal microbiome-related RPL, as well as some limitations to this intervention and prospective studies, are summarized.

## INTRODUCTION

The microbiome is a key factor that influences human health and disease in a variety of organs. While the gut microbiota has been extensively studied, other organs associated with microbiome medicine are now attracting attention. With the growing evidence for microbiome medicine, research into the vaginal microbiome is now gaining much ground with scientific evidence. Of note, this dynamic and intricate microecosystem changes microbiome composition due to menstrual life cycles and other environmental factors. To maintain a healthy vaginal microbiome, *Lactobacillus* with its antimicrobial products should dominate the reduced diversity. Moreover, these products released by *Lactobacillus* may prevent bacterial vaginosis (BV), which is a decline in vaginal commensals, including *Lactobacillus*. Studies show that this decline is associated with an influx of anaerobic microbes in the presence of lactic acid, which could have been utilized by *Lactobacillus*. Globally, BV is commonly associated with women of reproductive age and can lead to adverse gynecological and obstetric issues such as pelvic inflammatory disease, sexually transmitted diseases, and preterm birth. Among the BV patients, *Gardnerella vaginalis*, predominantly observed due to its polymicrobial biofilm properties, shelters other pathogens, thereby promoting the vaginal pathogen influx (1, 2).

As the vaginal microbiome is equally important to the health of the organ, its association with vaginal health is being explored and established based on specific taxa. This helps in identifying biomarkers that can predict any potential alterations. Even more interesting, however, is the recent research in recurrent pregnancy loss (RPL), and the vaginal microbiome is even more interesting (3). Another health benefit associated with the vaginal microbiome is seen in babies born through the vaginal canal without developing a risk of asthma during early life. Despite vaginal seeding, the gut microbiota of the infants born through cesarean delivery are still at risk of allergies, highlighting the importance of a healthy vaginal microbiome (4). In cows, a shift in the uterine microbiome with metritis is seen associated with pregnancy outcomes, especially at first insemination (5). Moreover, the increased obligate anaerobic, facultative bacteria and vaginal biofilm formation are typical of BV-associated *Gardnerella* spp. dominance. This vaginal biofilm may lead to ascending gynecological issues such as pregnancy-related infections, infertility, and preterm birth. Mechanistically, epithelial homeostasis induced by biofilm is altered, thereby promoting co-infections with sexually transmitted pathogens. This is exacerbated by the ineffectiveness of standard antibiotic therapy against these formed biofilms, leading to recurrent infections as “bacterial vaginosis syndrome” (6). Both the taxa and their physiological functions are crucial in determining BV-associated RPL (7). Although there are very important studies with clinically based evidence in medicine, a summary of these findings to make some meaningful biomarkers while suggesting prospective studies from some limitations is hopeful to make strong recommendations from this mini review.

## VAGINAL MICROBIOME AS A BIOMARKER FOR RPL

Microbiome sampling of cervicovaginal specimens revealed *Delftia* and other unknown bacteria as a typical RPL phenotype. In addition, *Microbacterium, Anaerobacillus, Chloroplast,* and *Streptococcus* are prevalent in chorioamnionitis. Moreover, *Anaerobacillus* and *Cutibacterium* are typical bacterial biomarkers in women who eventually miscarry. In a sense, the cervicovaginal specimen is crucial for predicting the risk or causes of RPL, as the presence or absence of certain bacteria in this specimen is a biological indicator (8). Cervicovaginal specimens include bacteria, mucus, and metabolites present in the vaginal canal. With vaginal microbiota transplantation, successful pregnancy and delivery are achievable after a number of stillbirths or late pregnancy losses. However, the potential donor must be thoroughly screened for sexually transmitted diseases, and specifications must be determined using an *in vitro* microbiome competition assay (9).

In addition to reduced *Lactobacillus*, reduced lactic acid-producing bacteria and *Bifidobacterium* are typical vaginal secretion microbiota in women with chronic endometritis. These patients have a high abundance of *Enterococcus, Streptococcus, Bifidobacterium,* and *Atopobium* (10). The *Herpesviridae* family, specifically Epstein-Barr virus, is a biomarker for preterm birth from the microbiome of both the cervical canal and the uterine cavity. This comes from a study that revealed a strong association between RPL and chronic infection, noting preterm premature rupture of fetal membranes as a complication in addition to prolonged oligohydramnios (11). In addition to the reduced diversity and richness, reduced prevalence of *Ureaplasma* and *Mycoplasma,* with *Lactobacillus* abundance as an indicator of a healthy pregnancy, will help to predict any associated adverse pregnancy outcomes (12). *Gardnerella vaginalis*, but not *Lactobacillus crispatus*, is abundant in the RPL group when vaginal microbiome samples are taken. Moreover, fungi are abundantly present in vaginal endometrial samples, confirming that the vaginal microbiome is associated with RPL (13).

**Fig 1:**
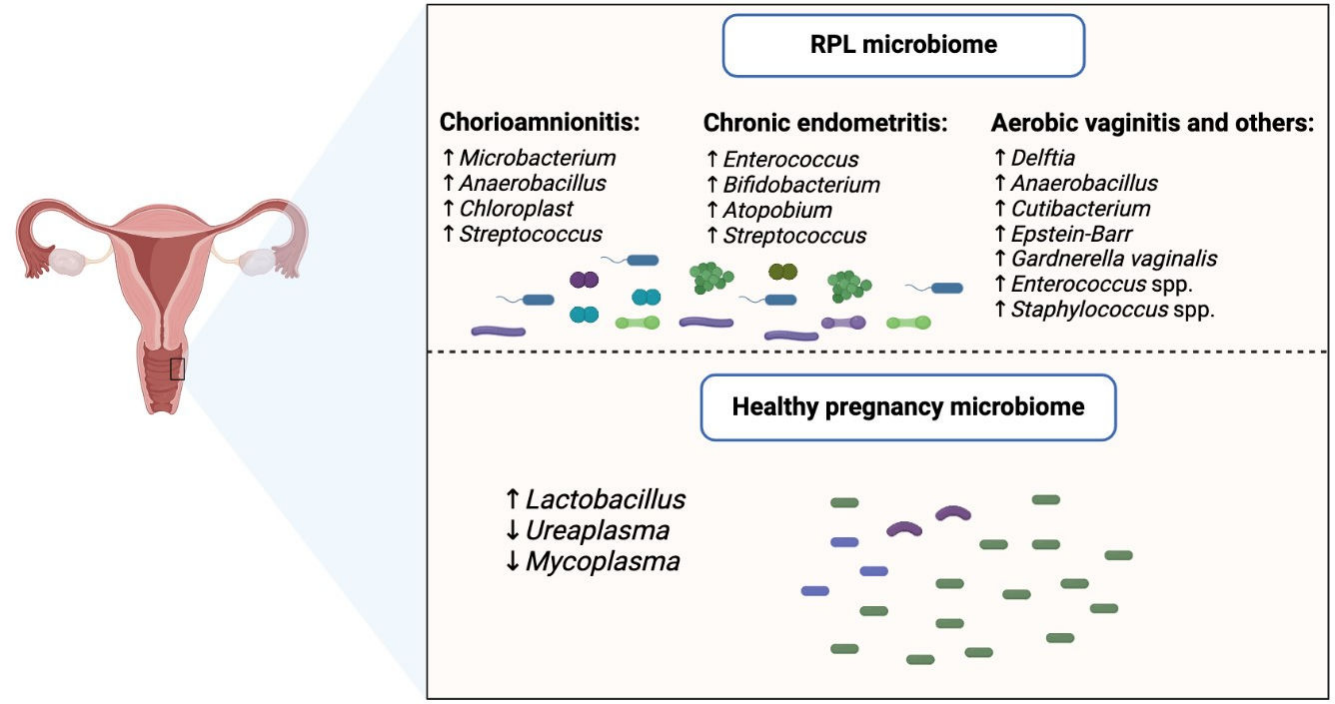
Vaginal microbiome as a biomarker for RPL.

Korean women had anaerobic vaginal species, which may indicate a high risk of pregnancy loss and may result in preterm birth. Moreover, *Lactobacillus crispatus*, but not *Lactobacillus iners,* dominated the vaginal microbes associated with women with miscarriage, whereas the latter is a typical biomarker for term pregnancy (14). In addition, *Lactobacillus iners*, but not *Lactobacillus acidophilus*, is present in both vaginal and endometrial microbiota samples from women with RPL (15). There is evidence that *Lactobacillus iners* may favor the growth of other vaginal microbiome pathogens and thus complicate pregnancy. The quest to affirm *Lactobacillus crispatus* as a quintessential health-associated commensal will only hold if both inflammation and reduced vaginal diversity are observed. In addition, this species must not be tampered with during the gestation period and the health of the pregnant woman (16). The microbiome associated with unexplained aerobic vaginitis is typically found in RPL, and the most prevalent species are *Enterococcus* spp. and *Staphylococcus* spp. This aerobic vaginitis is associated with low pH and enriched biofilm, which is strongly associated with multidrug resistance (17) (Fig. 1).

## IMMUNE RESPONSE AND METABOLITE CHANGES IN VAGINAL MICROBIOMERELATED RPL

As pregnancy progresses, the reduced diversity of the vaginal microbiome with a characteristic of *Lactobacillus* is seen with a shift in vaginal metabolites. These metabolites, notably acetate, alcohols, propionate, and biogenic amines, are typical dysbiotic-associated metabolites. Moreover, at the end of the healthy pregnancy, healthy vaginal metabolites, such as lactate, phenylalanine, glycine, leucine, and isoleucine, are highly prevalent. The progressive vaginal leukocytes are associated with IL-6 and IL-8, and these are seen with vaginal symptoms. For instance, increased IL-8 is a predictor of vaginal *Candida* spp., which is a typical vaginal pathogen that is positively correlated with O-acetylcholine, glucose, choline, and 4-hydroxyphenyllactate levels. Moreover, glycine, serine, and lactate are negatively correlated with cytokine concentration (18).

*Mobiluncus mulieris* in BV can significantly increase TNF-α, IL-6, IL-8, and MCP-1 with changes in metabolites such as succinate and nicotinamide, as well as oxidative stress markers such as cysteinylglycine disulfide, 2-hydroxyglutarate, and cysteinylglycine. In addition, vaginal infection with *Eggerthella* sp. can elevate glycerolipids and sphingolipids, which are pivotal for epithelial barrier function. Moreover, biogenic amines such as cadaverine and putrescine are associated with increased vaginal amine odor, elevated vaginal pH, and abnormal discharge (19). In case of embryonic miscarriage, heightened IL-10 and IL-2 levels correlate with increased abundances of *Lac_Roseburia, Fam_Finegoldia,* and *Lac_Coprococcus*, suggesting a direct link between immune response and microbiota shifts (20). Cervicovaginal specimen immune profiles have also established *Lactobacillus* spp. depleted vaginal microbiota, linking to pro-inflammatory cytokine levels (21). With the influx of pro-inflammatory cytokines, endometrial and vaginal dysbiosis are studied to compromise the receptivity and integrity of endometrial mucosa, thereby hampering a successful implantation of the embryo. While this evidence is promising to bring a paradigm shift in reproductive medicine, its established association with adverse obstetric outcomes, such as RPL and repeated implantation failure, warrants careful consideration. The pathophysiological mechanism involves a dysregulated inflammatory cascade within the vaginal and endometrial microenvironments, wherein immune cell activation has been identified as a hallmark feature of RPL (22). Multiple etiological factors have been implicated in triggering this aberrant immune response, including gram-negative bacteria components, fungal pathogens, viral infections, and neoplastic processes. Of particular significance, bacterial metabolites contribute to immune dysregulation through molecular mimicry mechanisms, thereby potentiating pathological immune activation. Notably, gut microbiota may promote systemic inflammation via the recruitment and expansion of pro-inflammatory lymphocytes (Th1/Th12 subsets), concomitant suppression of tolerogenic NK cells and regulatory T cells (Tregs), and subsequent disruption of fetomaternal immune tolerance. Intriguingly, certain commensal microbiota, particularly *Lactobacillus* spp., demonstrate remarkable immunomodulatory potential capable of counteracting these deleterious effects. Compelling experimental evidence indicates that vaginal transplantation of *Lactobacillus crispatus* significantly enhances immunotolerant responses at the maternal-fetal interface, thereby improving pregnancy outcomes (23).

## LIMITATIONS IN VAGINAL MICROBIOME ASSOCIATED WITH RPL

Although there are numerous breakthroughs and advanced bioinformatics tools to give robust analysis, human vaginal microbiome medicine faces persistent challenges in selecting optimal sequencing technology. This stems from ongoing methodological debates regarding conflicting analytical approaches (24). In addition, vaginal dysbiosis does not significantly influence the birth rate even though it poses a risk for early pregnancy loss (25). Moreover, fetal loss in the first trimester and its correlation with preterm birth rates with *in vitro* fertilization is not with significant evidence (26). There is an association between preterm rupture of membranes and BV; however, there are discrepancies regarding BV and pregnancy loss, particularly in sub-Saharan Africa. This warrants further research into the environmental factors, such as climate variations and social status for women in response to BV-associated RPL (27). Moreover, the treatment regime, dosage, and duration for infertile couples could lead to altered vaginal microbiome, and thus may cause RPL (28).

## PROSPECTS IN VAGINAL MICROBIOME ON RPL

Interventions to explore, in addition to the vaginal microbiome, include the endometrial microbiota and its association with the local endometrial microenvironment. This is evident on the principle of organ proximity, as the vaginal and endometrial niche may explain RPL (29). As part of targeted interventions in reproductive medicine, microbiome medicine spanning gut microbiome, endometrial and vaginal microbiome hold promise for addressing RPL. Thus, understanding the complexities and specifics of this unexplained diagnosis will confer effective reproductive health outcomes while minimizing high risks (30). Vaginal microbiome screening could enable a predictive targeted therapeutic approach not only for RPL but also for preventing miscarriage (31). A large cohort study combining gut and vaginal microbiome analyses in cases of early pregnancy loss and complications may reveal significant microbiome-associated gynecological issues (32).

The self-esteem of women, often affected by stigma when visiting gynecologists, may improve if self-swab collection becomes an option. However, this should be collected from the lower vaginal area, as other vaginal regions may harbor distinct microbiomes, thereby altering the sensitivity of the results (33). For instance, the cervix uteri microbiome holds promise for prognosis and also to prevent early miscarriages as part of RPL (34). Moreover, vaginal hydrogen peroxide-producing *Lactobacillus* and fastidious BV-associated bacteria increase the risk of pelvic inflammatory disease, which may contribute to RPL (35).

Unlike gut microbiota, increased diversity in the vaginal microbiome is not always indicative of health. It was believed that oral contraceptives or levonorgestrel intrauterine systems were associated with microbiome composition. However, the increased diversity in the vaginal microbiome is prominent during the menstrual cycle and subsequently reduced *Lactobacillus* spp. Metabolite changes, cytokines, and the menstrual cycle may modulate the transitional changes to the vaginal bacterial communities and even sub-communities. A *Lactobacillus*-dominant vaginal ecosystem confers significant biological and clinical benefits for pregnant women (36, 37).

As an indicator of follicular and luteal phases, this diversity is correlated with serum estradiol levels (38). Among the physiological changes associated with the vaginal microbiome is maternal stress, and this may predispose offspring to neurodevelopmental disorders. Moreover, the neonate gut microbiota alterations induce metabolite profile alterations such as amino acid and, to some extent, energy balance shift. However, questions still persist if maternal stress is a causation to maternal vaginal protein that is associated with vaginal immunity. Although a reduced vaginal *Lactobacillus* is reflecting on offspring alteration for this genus, it is not clear if this reduction is as a result of maternal stress or the stress causing the reduction (39).

**Fig 2:**
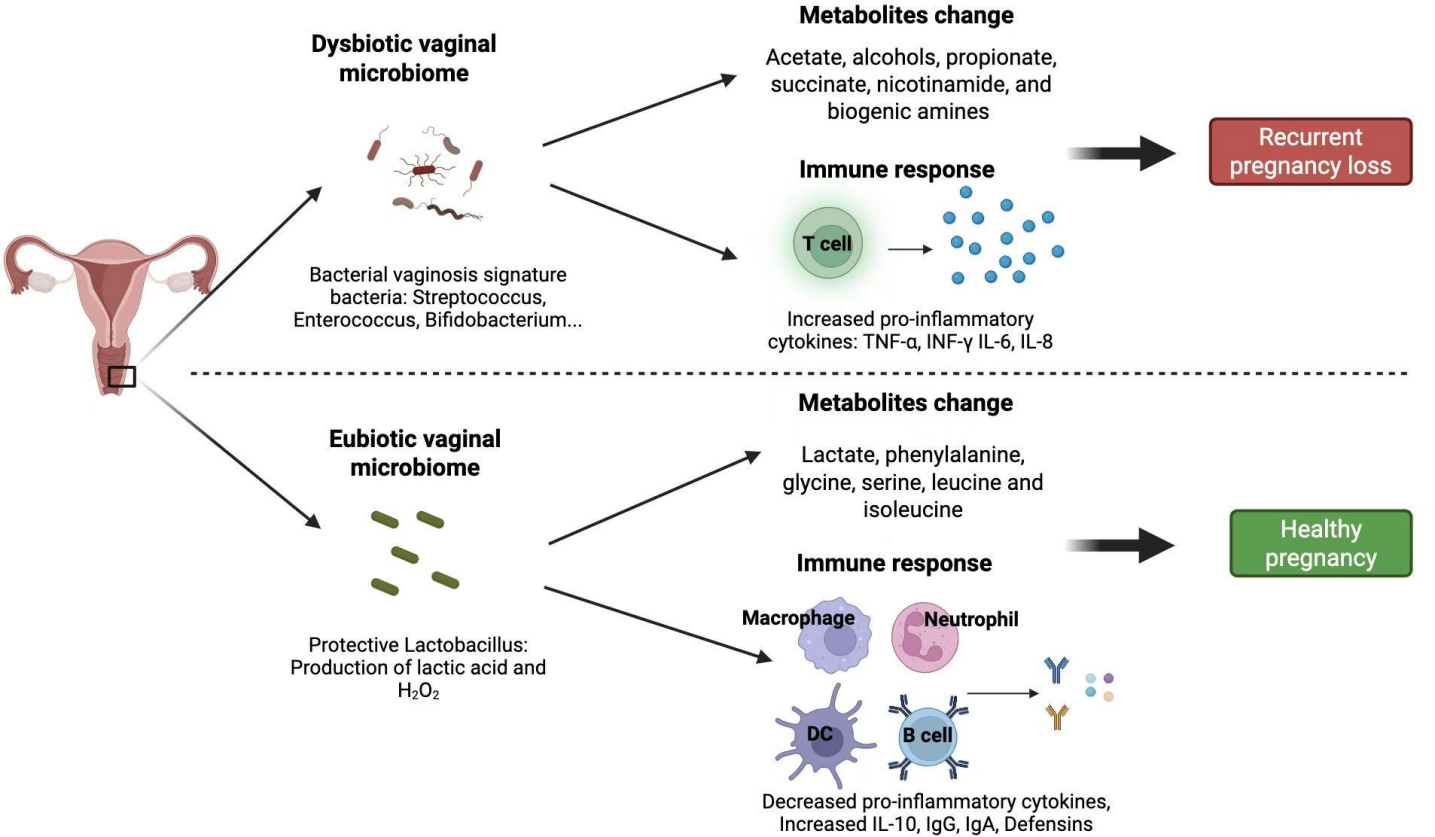
Immune response, metabolite changes, limitations, and prospects in vaginal microbiome-associated with RPL.

In determining the vaginal microbiome to RPL, the analysis used may determine a lot. For instance, while Nugent score could not give any meaningful physiological changes, Bayesian network score revealed vaginal pH as associated with *Gardnerella, Eggerthella, Ruminococcaceae*, *Sneathia,* and *Dialister*, while vaginal itch, irritation, odor, yeast infection, and discharge are associated with *Corynebacterium*, *Proteobacteria,* and *Lactobacillus jensenii*. Moreover, none of ethnicity, age, or previous pregnancy is directly related to clinical BV (40, 41) (Fig. 2).

## CONCLUSION

The association or correlation of vaginal microbiome imbalance and RPL will require further studies that must see a large human cohort as a clinical trial to give much scientific grounds to the existing knowledge. For instance, each patient or woman will experience a distinct microbiome compositional alteration throughout the period of pregnancy. Since it will be cumbersome to ascertain the specific metabolites shift and immune response due to this dysbiotic vaginal microbiome, a uniform taxon in addition to increased lactobacilli as biomarkers is required. Moreover, the study participants should be screened before taking part in the trial and must ensure that other environmental factors, as well as genetic factors, are known prior to specimen analysis.
